# Does ^18^F-Fluorocholine PET/CT add value to positive parathyroid scintigraphy in the presurgical assessment of primary hyperparathyroidism?

**DOI:** 10.3389/fmed.2023.1148287

**Published:** 2023-04-27

**Authors:** Alessio Imperiale, Jacob Bani, Gianluca Bottoni, Adrien Latgé, Céline Heimburger, Ugo Catrambone, Michel Vix, Giorgio Treglia, Arnoldo Piccardo

**Affiliations:** ^1^Nuclear Medicine and Molecular Imaging, Institut de Cancérologie de Strasbourg Europe (ICANS), Strasbourg University Hospitals, Strasbourg, France; ^2^Molecular Imaging, DRHIM, Institut Pluridisciplinaire Hubert Curien (IPHC), UMR7178, CNRS, University of Strasbourg, Strasbourg, France; ^3^Nuclear Medicine, Ente Ospedaliero "Ospedali Galliera", Genoa, Italy; ^4^Nuclear Medicine, Hopital Civil de Haguenau, Haguenau, France; ^5^General Surgery, Ente Ospedaliero "Ospedali Galliera“, Genoa, Italy; ^6^General, Digestive, and Endocrine Surgery, IRCAD-IHU, Strasbourg University Hospitals, Strasbourg, France; ^7^Clinic for Nuclear Medicine, Imaging Institute of Southern Switzerland, Ente Ospedaliero Cantonale, Bellinzona, Switzerland; ^8^Nuclear Medicine and Molecular Imaging, Lausanne University Hospital, Lausanne, Switzerland; ^9^Faculty of Biology and Medicine, University of Lausanne, Lausanne, Switzerland; ^10^Faculty of Biomedical Sciences, Università Della Svizzera Italiana, Lugano, Switzerland

**Keywords:** hyperparathyroidism, parathyroid, PET, choline, scintigraphy, sestamibi, parathyroid adenoma

## Abstract

**Introduction:**

To investigate the value of presurgical ^18^F-FCH PET/CT in detecting additional hyperfunctioning parathyroids despite a positive ^99m^Tc-sestamibi parathyroid scintigraphy in patients with primary hyperparathyroidism (pHPT).

**Methods:**

This is a retrospective study involving patients with pHPT, positive parathyroid scintigraphy performed before ^18^F-FCH PET/CT, and parathyroid surgery achieved after PET/CT. Imaging procedures were performed according to the EANM practice guidelines. Images were qualitatively interpreted as positive or negative. The number of pathological findings, their topography, and ectopic location were recorded. Histopathology, Miami criterion, and biological follow-up were considered to ensure effective parathyroidectomy confirming the complete excision of all hyperfunctioning glands. The impact of ^18^F-FCH PET/CT on therapeutic strategy was recorded.

**Results:**

64/632 scanned pHPT patients (10%) were included in the analysis. According to a per lesion-based analysis, sensitivity, specificity, positive predictive value, and negative predictive value of ^99m^Tc-sestamibi scintigraphy were 82, 95, 87, and 93%, respectively. The same values for ^18^F-FCH PET/CT were 93, 99, 99, and 97%, respectively. ^18^F-FCH PET/CT showed a significantly higher global accuracy than ^99m^Tc-sestamibi scintigraphy: 98% (CI: 95–99) vs. 91% (CI: 87–94%). Youden Index was 0.79 and 0.92 for ^99m^Tc-sestamibi scintigraphy and ^18^F-FCH PET/CT, respectively. Scintigraphy and PET/CT were discordant in 13/64 (20%) patients (49 glands). ^18^F-FCH PET/CT identified nine pathologic parathyroids not detected by ^99m^Tc-sestamibi scintigraphy in 8 patients (12.5%). Moreover, ^18^F-FCH PET/CT allowed the reconsideration of false-positive scintigraphic diagnosis (scinti+/PET-) for 8 parathyroids in 7 patients (11%). The ^18^F-FCH PET/CT influenced the surgical strategy in 7 cases (11% of the study population).

**Conclusion:**

In a preoperative setting, ^18^F-FCH PET/CT seems more accurate and useful than ^99m^Tc-sestamibi scan in pHPT patients with positive scintigraphic results. Positive parathyroid scintigraphy could be not satisfactory before neck surgery particularly in patients with multiglandular disease, suggesting a need to evolve the practice and define new preoperative imaging algorithms including ^18^F-FCH PET/CT at the fore-front in pHPT patients.

## Introduction

Primary hyperparathyroidism (pHPT) is a common endocrine disease caused by excessive secretion of parathyroid hormone (PTH) from one or more hyperfunctioning parathyroid glands. In 80% of cases, pHPT is related to a solitary parathyroid adenoma. Moreover, the patient may harbor multiple-gland disease (MGD), with double adenomas or 4-gland hyperplasia in 15–20% of cases (1), and ectopic hyperfunctioning parathyroids in about 16% of cases (2).

A mini-invasive parathyroidectomy (MIP) is emerging as the standard of care in patients with pHPT, reducing the operative complications, time, and cost with cure rates comparable to those of surgery (3). After a decision was made to proceed with parathyroidectomy, the success of the minimally invasive surgical strategy depends on effective detection of the hyperfunctioning gland during the preoperative work-up (4, 5). Currently, neck ultrasound (US) and parathyroid scintigraphy with ^99m^Tc-sestamibi are recommended as first-line imaging techniques. However, US diagnostic performances are influenced by the experience of the operator, and scintigraphy success largely depends on the adopted scintigraphic protocol (6, 7), and on patient underlying pathology. Indeed, the systematic review by Ruda et al. (8) alerts about the low sensitivity of ^99m^Tc-sestamibi scintigraphy for the detection of multigland hyperplasia (44%) and double adenomas (29.9%).

In recent years, positron emission tomography/computed tomography/computed tomography (PET/CT) with ^18^F-fluorocholine (^18^F-FCH) has emerged as second-line imaging with high resolution, low radiation exposure, and shorter examination times in patients with previously negative or inconclusive ^99m^Tc-sestamibi scintigraphy and/or ultrasound (9). A growing body of evidence suggests the high sensitivity of ^18^F-FCH PET/CT in detecting hyperfunctioning parathyroids (sensitivity and detection rate up to 90 and 80%, respectively) in patients with pHPT (10), even in reoperated patients (11). Recently published parathyroid imaging guidelines suggest, when possible, ^18^F-FCH PET/CT as a potential “alternative” first-line option in patients with pHPT (12). All these considerations emphasize the debate about the possible role of ^18^F-FCH PET/CT as first-line nuclear medicine imaging method for preoperative localization of hyperfunctioning parathyroids in patients with pHPT (13). With a perspective to participate in the discussion, in the present study we analyzed whether and in what percentage of patients with pHPT scheduled for neck surgery, ^18^F-FCH PET/CT detect additional hyperfunctioning parathyroids despite a positive ^99m^Tc-sestamibi parathyroid scintigraphy. The impact of ^18^F-FCH PET/CT results on therapeutic strategy has been also investigated.

## Materials and methods

### Patient population

This is a non-interventional retrospective study involving patients with HPT who underwent ^18^F-FCH PET/CT for preoperative identification of hyperfunctioning parathyroid glands at the nuclear medicine unit of the *Strasbourg University Hospital/*Institut de Cancérologie de Strasbourg Europe (ICANS) of Strasbourg, France, and the Galliera Hospitals of Genoa, Italy. 632 patients were retrieved from own Institutional parathyroid ^18^F-FCH PET/CT registry from March 2016 to March 2021. Only patients satisfying the following criteria were retrospectively included: (1) pHPT, (2) positive parathyroid scintigraphy performed before ^18^F-FCH PET/CT, (3) parathyroid surgery achieved after ^18^F-FCH PET/CT.

Clinical, imaging and biological data were extracted from hospital databases, clinician reports, and biomedical laboratories, including sex, age, previous parathyroid surgery, the results of diagnostic imaging performed before ^18^F-FCH PET/CT [i.e., neck ultrasound (US)], patient genetic status, calcimimetic treatment, hypercalcemia-related symptoms, PTH, and calcium serum concentrations. Surgical procedures, perioperative and post-surgical follow-up PTH measurements, and pathological reports concerning the parathyroid surgery after ^18^F-FCH PET/CT were collected.

In accordance with French Jarde Law, the *Ethical Committee of Strasbourg University Hospital and Faculty of Medicine* waives the requirement for informed consent for retrospective use of anonymized data obtained in the course of routine clinical care. The local Institutional Review Board *has confirmed that no ethical approval is required (CE-2022-100).*

### Imaging procedures

All scintigraphic and PET/CT procedures were achieved according to the EANM practice guidelines for parathyroid imaging (12). Parathyroid scintigraphy was performed using single-tracer (^99m^Tc-sestamibi) dual-phase technique, or dual-tracer subtraction technique (^99m^Tc-sestamibi/^123^I or ^99m^Tc-sestamibi/^99m^Tc-pertechnetate). Imaging protocol included neck and mediastinum anterior planar scan, followed by single photon emission tomography/computed tomography (SPECT/CT) acquisition. A low-dose CT scan was performed for anatomical correlation only. Concerning PET/CT, all examinations were performed by combined devices equipped with time-of-flight technology. Patients fasted for at least 6 h before the intravenous injection of about 2 MBq/kg of ^18^F-FCH. PET acquisition from the mandible to the carina was acquired 60 min after ^18^F-FCH administration in the supine position with arms along the body and headrest. PET datasets were reconstructed iteratively by ordered subset expectation maximization (OSEM) algorithm, using no contrast-enhanced CT for attenuation correction.

Scintigraphic and PET/CT images were qualitatively interpreted as positive or negative. Focal non-physiological uptake corresponding to any cervical or thoracic abnormalities discriminable from thyroid tissue and positioned in typical parathyroid sites or in ectopic areas was considered positive. The number of pathological findings on ^99m^Tc-sestamibi scintigraphy and ^18^F-FCH PET/CT, their topography in reference to the midline and the thyroid gland, ectopic location, and embryologic origin of the parathyroid (superior vs. inferior gland) were recorded.

### Gold standard

Histopathology after parathyroidectomy, associated with a decrease of more than 50% of perioperative PTH blood level (Miami criterion), AND/OR a 6-months biological follow-up were considered to ensure operative success confirming the complete excision of all hyperfunctioning parathyroid tissue (true positive results) (14). Intraoperative parathyroid hormone monitoring (IPM) was also used to preserve the normally functioning parathyroid glands during parathyroidectomy with resection of only the pathological. Normal glands (true negative results) were defined according to a combination of neck surgical exploration result, biological follow-up (PTH, serum calcium), and Miami “>50% intraoperative PTH drop” criterion.

^18^F-FCH PET/CT was considered contributory to modifying the therapeutic strategy according to the following situations: (1) lateralize the side of mini-invasive parathyroidectomy (MIP): right vs. left, or left vs. right, (2) bilateral MIP converted in unilateral MIP, (3) unilateral MIP converted in bilateral MIP, and (4) MIP converted in cervicotomy eventually associated to mediastinoscopy.

### Statistical analysis

Categorical variables were presented as numbers and percentages. Results for continuous data were expressed as mean ± standard deviation, or median and interquartile (IQ) range, as appropriate. Diagnostic performance of both ^18^F-FCH PET/CT and ^99m^Tc-sestamibi scintigraphy in detecting hyperfunctioning parathyroids were assessed on a per-lesion basis. Sensitivity (Se), specificity (Sp), positive predictive value (PPV), negative predictive value (NPV), and overall accuracy were assessed and presented with 95% confidence intervals (95% CI). Finally, the Youden Index, which is independent from the prevalence of the disease, was estimated as (Sp + Se)-1. A *p* value <0.05 were considered statistically significant. Statistical analysis has been provided using Graphpad Prism software (v.9.1.1, 2021).

## Results

### Patient population

In the study period, 64 among 632 scanned patients (10%) were addressed for preoperative ^18^F-FCH PET/CT investigation after a positive ^99m^Tc-sestamibi scintigraphy, and were included in the analysis. The median time interval between a positive ^99m^Tc-sestamibi scintigraphy and ^18^F-FCH PET/CT was 104 days (IQ range: 47–247). Patients’ refusal immediate parathyroidectomy, or delayed surgery due to the COVID-19 pandemic, mainly explain why patients underwent ^18^F-FCH PET/CT despite a positive ^99m^Tc-sestamibi scintigraphy, and the delay between scintigraphy and PET/CT. In all cases, cross-disciplinary team stated about ^18^F-FCH PET/CT indication. Medical treatment, if any, was not changed between the two examinations.

Population median age was 65 years (IQ: 58–70, range 29–85), with a female to male ratio of 3.6 (14:50). The preoperative median PTH and serum calcium levels were 116.3 ng/l (IQ range: 92.6–170.8) and 2.68 nmol/l (IQ range: 2.58–2.80), respectively. Five patients (8%) presented with recurrent/persistent pHPT, and the remaining 59 cases (92%) had no previous parathyroid surgery. Thus, a total of 248 glands (12 and 236, respectively) were considered for further analysis. Result from neck US was not available for 4 of 64 selected patients, including 1 case of primary diagnosis, 2 persistent pHPT, and 1 recurrent pHPT. US was positive in 36/60 patients (60%) and negative in the remaining 24 cases. No case of MGD was suspected by US. After ^18^F-FCH PET/CT, 40 patients underwent MIP (63%), 23 exploratory cervicotomy (36%), and 1 thoracoscopy (1%), for a total of 72 excised pathologic parathyroid glands corresponding to 56 adenomas and 16 hyperplasia. Size and weight of pathologic parathyroid was available in 67 and 44 glands, and the median values were 12 mm (IQ: 10–20) and 0.4 g (IQ: 0.2–1.0), respectively. Five patients had MGD (8%). Median time interval between ^18^F-FCH PET/CT and parathyroid surgery was 138 days (IQ range 63–271). Median biological follow-up after parathyroid surgery was 3 months (IQ range 1–12), and 53% of patients had at least 6 months post-surgical follow-up.

### ^99m^Tc-sestamibi scintigraphy

Parathyroid scintigraphy was performed using ^99m^Tc-sestamibi dual-phase technique in 5 patient (8%), and dual-tracer subtraction technique including ^99m^Tc-sestamibi/^123^I or ^99m^Tc-sestamibi/^99m^Tc-pertechnetate in 59 patients (92%). According to the inclusion criteria, parathyroid scintigraphy was positive in all patients, suggesting the presence of one or two hyperfunctioning parathyroids in 60 and 2 patients, respectively. According to a per lesion-based analysis, parathyroid scintigraphy was true-positive in 59 cases (24%), false-positive in 9 cases (4%), true-negative in 167 cases (68%), and false-negative in 13 cases (5%, 5 adenomas, 8 hyperplasia). Se, Sp, PPV, NPV, and global accuracy of ^99m^Tc-sestamibi scintigraphy were 82, 95, 87, 93, and 91%, respectively. The Youden index was 0.79 (Table 1).

**Table 1:** Head-to-head comparison (per gland analysis) of diagnostic performances of ^99m^Tc-sestamibi scintigraphy and ^18^F-FCH PET/CT in the whole population of 64 patients with pHPT.

	TP	FP	TN	FN	Se (%)	Sp (%)	PPV (%)	NPV (%)	Global accuracy (%)	Youden index
^99m^Tc-sestamibi scintigraphy	59	9	167	13	82 (71–90)	95 (91–98)	87 (77–93)	93 (89–95)	91 (87–94)	0.79
^18^F-FCH PET/CT	67	1	175	5	93 (85–98)	99 (97–100)	99 (90–100)	97 (94–99)	98 (95–99)	0.92

TP, true-positive; FP, false-positive; TN, true-negative; FN, false-negative; Se, Sensitivity; Sp, specificity; PPV, positive predictive value; NPV, negative predictive value. In parentheses are reported the 95% CI values.

### ^18^F-FCH PET/CT

^18^F-FCH PET/CT was positive in 63 out of 64 included patients indicating the presence of one, two, or three hyperfunctioning parathyroids in 57, 5, and 1 patient, for a total of 70 pathological parathyroids. According to a per lesion-based analysis, ^18^F-FCH PET/CT was true-positive in 67 cases (27%), false-positive in 3 cases (1%), true-negative in 175 cases (71%), and false-negative in 1 case. Se, Sp, PPV, NPV, and global accuracy of ^18^F-FCH PET/CT were 93, 99, 99, 97, and 98%, respectively. Although higher values of Se, Sp, PPV, NPV of ^18^F-FCH PET/CT, no statistically significance was reached probably due to the limited number of analyzed glands. The Youden index was 0.92 (Table 1).

### ^18^F-FCH PET/CT versus ^99m^Tc-sestamibi scintigraphy

^99m^Tc-sestamibi scintigraphy and ^18^F-FCH PET/CT showed concordant results in 51/64 (80%) patients (199 glands). In a per lesion-based analysis, ^99m^Tc-sestamibi scintigraphy and ^18^F-FCH PET/CT were concordant positive in 51/199 glands (26%) and concordant negative in 148/199 glands (74%). The remaining 13/64 (20%) patients (49 glands) showed discordant imaging results: 7/49 glands were scinti-/PET+, and 5/49 glands were scinti+/PET- (Table 2). In patients with discordant results, median time interval between ^99m^Tc-sestamibi scintigraphy and ^18^F-FCH PET/CT was 161 days (IQ range 63–254). ^99m^Tc-sestamibi scintigraphy was true-positive in 9 glands, false-positive in 8, true-negative in 21, and false-negative in 11. On the other hand, ^18^F-FCH PET/CT was true-positive in 17 glands, false-positive in 2, true-negative in 27, and false-negative in 3. In the whole population, ^18^F-FCH PET/CT showed a significantly higher global accuracy (98% (CI: 95–99) vs. 91% (CI: 87–94%), *p* = 0.0001) and superior Youden Index (0.79 vs. 0.92) than ^99m^Tc-sestamibi scintigraphy.

**Table 2:** Characteristics and therapeutic management of 13 patients with discordant ^99m^Tc-sestamibi scintigraphy and ^18^F-FCH PET/CT findings.

Pt	Sex (M/F)	Age (y)	Genetic status	Cinacalcet (Y/N)	Serum PTH (ng/L)	Calcemia (mmol/L)	Parathyroid localization		Histology (from PET target)	Parathyroid		Surgical strategy before PET/CT	Surgical strategy after PET/CT	Modification of surgical strategy after PET/CT
							^99m^Tc-sestamibi scintigraphy	^18^F-FCH PET/CT		Height (mm)	Weight (g)			
1	F	65	/	N	232	3.4	LS, LI*	LS	Ad	20	1.0	Left MIP	Left MIP	No
2	M	63	/	Y	143	2.5	RS*	**LS**	Ad	15	na	Right MIP	Left MIP	Yes
3	F	58	/	N	148	2.5	RI	RI, **LI**	Hpl, Hpl	10, 10	1.0, 1.0	Unilateral MIP	bilateral MIP	Yes
4	F	62	/	N	243	2.9	LS	RI*, LS	Ad	10	1.0	Thyroidectomy for cancer suspicion	Thyroidectomy for cancer suspicion	No
5	M	79	/	Y	237	3.1	RI, LS*	RI	Ad	100	10.0	Thyroidectomy for goiter	Thyroidectomy for goiter	No
6	M	48	/	N	102	2.6	LS	not detected	na	na	na	Left MIP	No contributive result	No (*)
7	F	28	MEN1	N	133	2.7	LI	**LS**, **RI**, LI	Hpl, Hpl, Hpl	na	na	Left MIP	Cervicotomy	Yes
8	F	56	/	N	72	2.3	LS	LS, **ectopic (mediastinum)**	Hpl, Hpl	12, na	na, na	Left MIP	Cervico-thoracotomy	Yes
9	F	72	/	N	44	2.6	Intrathyroidal*	Intrathyroidal*, **LI**	Ad	10	0.4	Thyroidectomy for cancer suspicion	Thyroidectomy for cancer suspicion	No
10	M	68	/	N	174	2.9	RI*, LS*	**LI**	Ad	13	0.7	Bilateral MIP	Left MIP	Yes
11	F	68	/	N	199	2.6	RI, LI*	RI	Ad	10	0.1	Bilateral MIP	Right MIP	Yes
12	F	62	/	N	49	2.7	RI	RI, **LS**	Hpl, Hpl	10, 10	0.1, 0.1	Right MIP	Cervicotomy	Yes
13	F	46	/	N	70	2.6	RS*	**RI**	Ad	8	0.1	Right MIP	Right MIP	No

**Figure 1:**
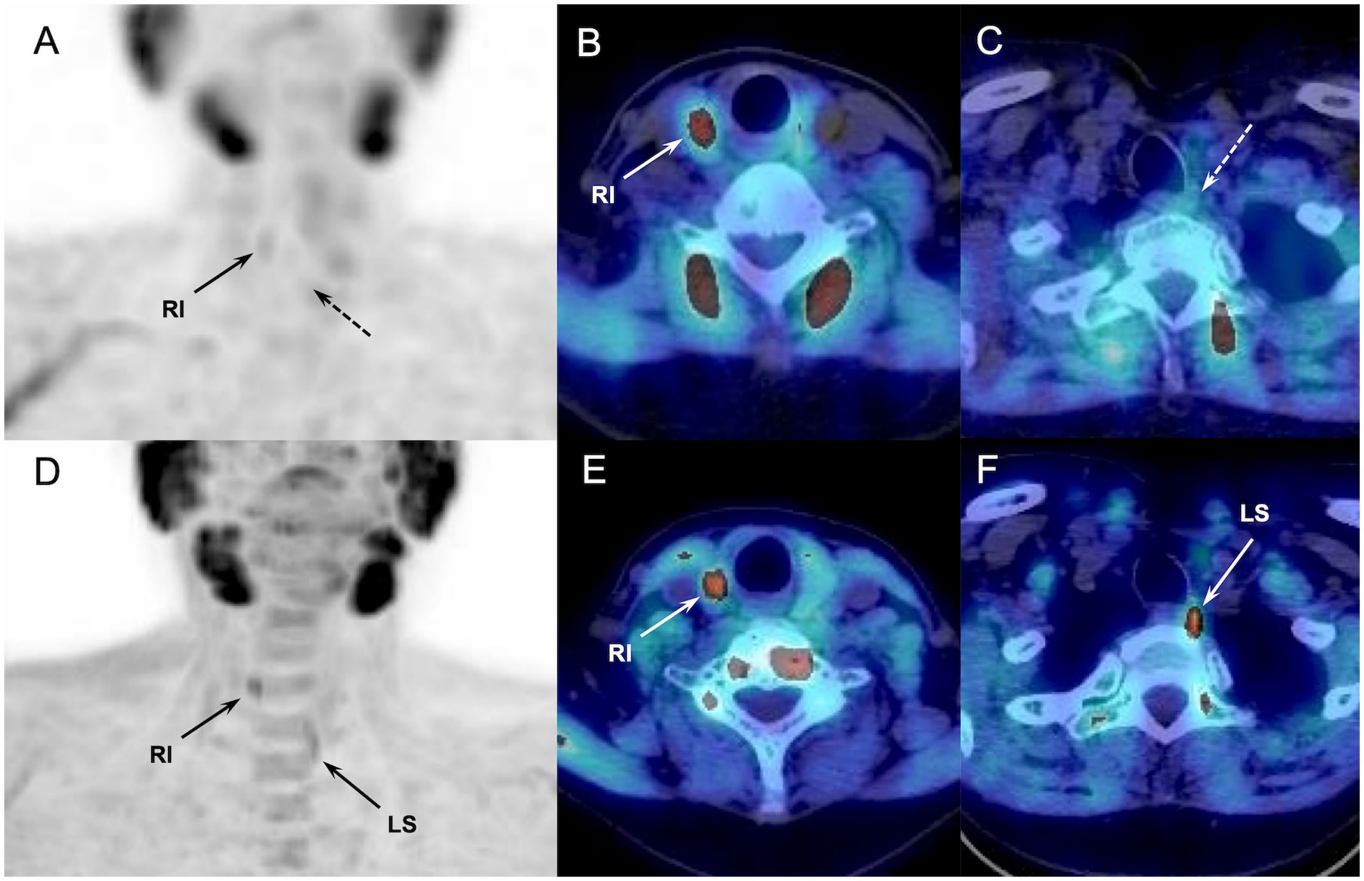
Discordant results of ^99m^Tc-sestamibi parathyroid scintigraphy (upper panel, A-C) and ^18^F-FCH PET/CT (lower panel, D-F) (anterior MIP, coronal and axial fusion images) performed in a 62-y-old MEN patient with sporadic pHPT at primary staging (patient#12, Table 2). ^18^F-FCH PET/CT revealed a hyperfunctioning right inferior and left superior (in lower position) parathyroids (arrows), confirmed after surgery. ^99m^Tc-sestamibi scintigraphy detected only the right inferior gland.

**Figure 2:**
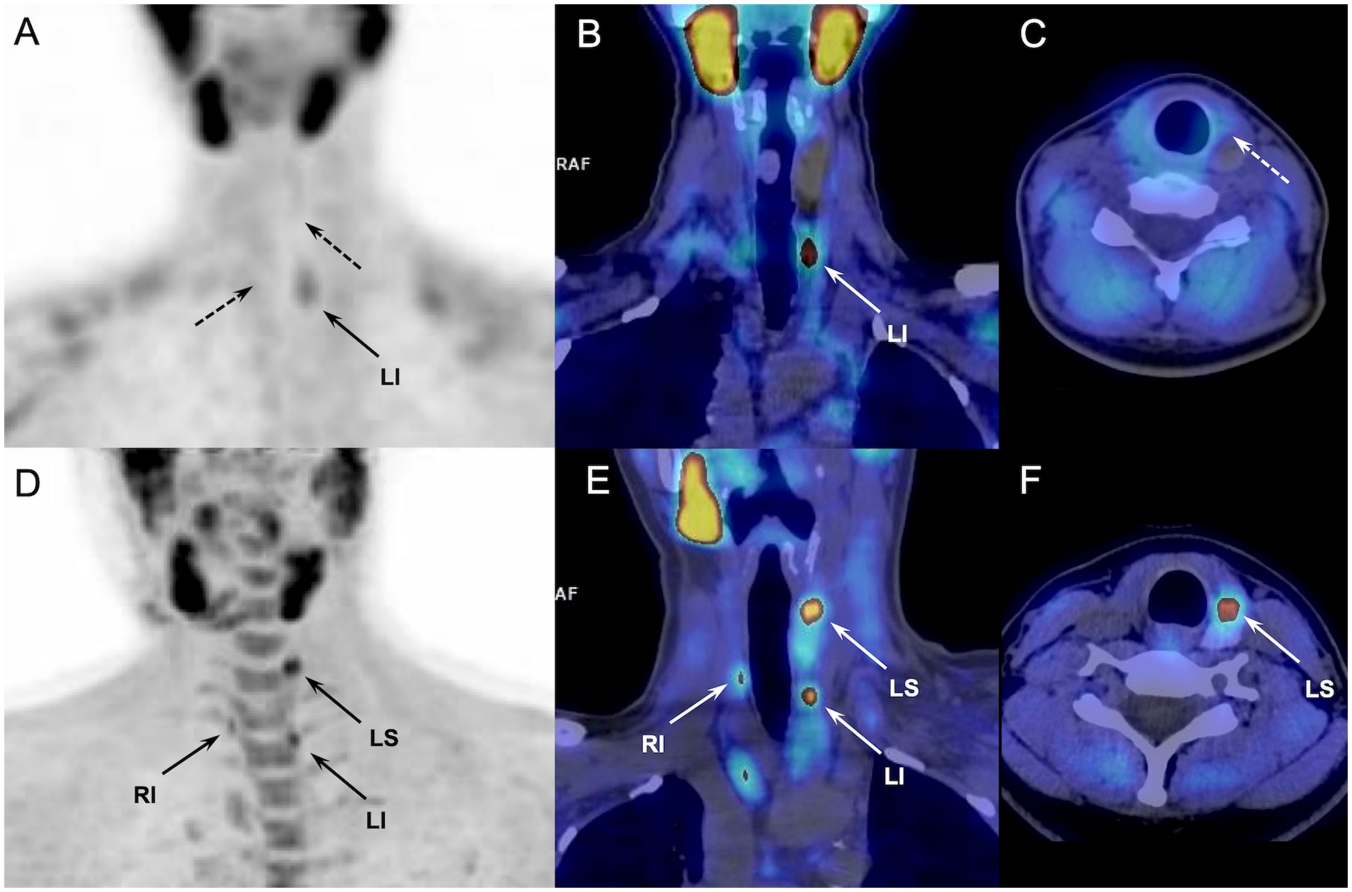
Discordant results of ^99m^Tc-sestamibi parathyroid scintigraphy (upper panel, A-C) and ^18^F-FCH PET/CT (lower panel, D-F) (anterior MIP, coronal and axial fusion images) performed in a 28-y-old MEN1 patient with previous history of superior right parathyroidectomy (patient#7, Table 2). ^99m^Tc-sestamibi scan showed a pathological left inferior parathyroid (arrow). ^18^F-FCH PET/CT confirmed scintigraphy findings and detected 2 more hyperfunctioning glands (arrows, inferior right and superior left) not revealed by scintigraphy, and afterwards confirmed by pathology after surgical excision.

Pt, patient; LS, left superior; LI, left inferior; RS, right superior; RI, right inferior, *False positive results. In Bold are indicated the nine pathologic parathyroids identified by ^18^F-FCH PET/CT and not detected by ^99m^Tc-sestamibi scintigraphy, Ad, adenoma; Hpl, hyperplasia; MIP, mini-invasive parathyroidectomy, (*): the only case of persistent pHPT after surgery. Patients #7 and #12 are represented in Figures 1, 2.

^18^F-FCH PET/CT identified nine pathologic parathyroids not detected by ^99m^Tc-sestamibi scintigraphy in 8 out of 64 examined patients (12.5%) (Figure 1), allowing to detect four patients with MGD, and 1 ectopic gland. In all eight patients, parathyroid scintigraphy was performed according to the ^99m^Tc-sestamibi/^123^I subtraction protocol. Moreover, ^18^F-FCH PET/CT allowed the reassessment of false-positive scintigraphic diagnosis (scinti+/PET-) for 8 parathyroids from 7/64 patients (11%). Only one hyperfunctioning gland in one patient was missed on ^18^F-FCH PET/CT and correctly identified by ^99m^Tc-sestamibi scintigraphy. Neither pathological features nor hormone secretion or the patient’s clinical profile explained the discordant imaging results.

In two of the six patients with recurrent/persistent pHPT, ^18^F-FCH PET/CT revealed three hyperfunctioning glands undetected by ^99m^Tc-sestamibi scintigraphy and confirmed as glandular hyperplasia after surgical excision. One patient was MEN1. Both patients showed no biological abnormalities more than 6 months later. An additional case with recurrent pHPT showed a scinti+/PET- imaging pattern. This patient presented persistent disease after a second surgery and removal (hyperplasia) of the parathyroid indicated by scintigraphy.

### Patient management

In the 13 patients with discordant imaging results, the change of patient management was discussed for each case with the clinical and surgical investigators involved in the decision-making process. Accordingly, the preoperative ^18^F-FCH PET/CT was considered contributory to influence the surgical strategy in 7 patients (54%) (Table 2). In those cases, ^18^F-FCH PET/CT allowed the change of the side of minimally invasive surgical approach (MIP) in 1 patient, the conversion of bilateral MIP in unilateral MIP in 2 patients (3), the conversion of unilateral MIP in bilateral MIP in 1 patient, and the conversion of MIP in cervicotomy/thoracotomy in 3 patients.

## Discussion

The finding from our study confirms the diagnostic relevance of ^18^F-FCH PET/CT in pHPT, emerging as an efficient imaging option for preoperative detection of hyperfunctioning parathyroids. If previous studies emphasized the usefulness of ^18^F-FCH PET/CT in patients with negative or inconclusive ultrasound and/or ^99m^Tc-sestamibi scintigraphy (9, 15–18), our results bring new and, in our opinion, important elements for discussion because they specifically concern selected patients with positive ^99m^Tc-sestamibi scintigraphy. To our knowledge, no data are available about the potential benefit of ^18^F-FCH PET/CT in terms of preoperative detection of additional hyperfunctioning parathyroids in this specific clinical scenario. Herein, we highlight the diagnostic and therapeutic impact of preoperative ^18^F-FCH PET/CT allowing the identification of nine pathologic parathyroids in 12.5% of patients not previously detected by ^99m^Tc-sestamibi scintigraphy, and confirmed after parathyroidectomy. Moreover, ^18^F-FCH PET/CT influenced the surgical management of 7 patients, equivalent to 11% of the study population.

Surgical success requires an experienced surgeon and depends on the effective identification of the hyperfunctioning gland during the preoperative work-up (4, 5), especially in view of a conservative surgical strategy. MIP can necessitate conversion to bilateral surgery in about 20% of cases (19), mostly because of incorrect preoperative parathyroid localization and unrecognized MGD. Therefore, there is no real reason to discard the use of ^18^F-FCH PET/CT as first-line imaging in order to reduce the need for repeat surgery, which is commonly expensive and with increased morbidity. After deciding to proceed with parathyroidectomy, the choice of the most appropriate diagnostic imaging modality to guide gland localization should rely on diagnostic performance. Of course, among the various techniques proposed for the detection and treatment of hyperfunctioning glands, the adopted method which must be available, safe, and inexpensive. ^99m^Tc-sestamibi scintigraphy is the current investigation for presurgical detection of hyperfunctioning parathyroid glands in pHPT patients (20). However, its sensitivity is suboptimal and significantly lower in MGD compared with single-gland disease (21, 22). This data has some important consequences because the effectiveness of MIP relies on the success in identifying patients with MGD and ectopic glands that cannot be visualized with US. Thus, it is generally patients with hyperplasic glands, smaller and more difficult to detect, who may benefit most from the use of ^18^F-FCH PET/CT. In this regard, pHPT patients with multiple endocrine neoplasia of type-1 (MEN1) represent critical clinical candidates for accurate parathyroid diagnostic imaging with potential therapeutic implications (Figure 2). In this setting only few data are available comparing the imaging findings of parathyroid US, ^99m^Tc-sestamibi scintigraphy, and ^18^F-FCH PET/CT. In a recent retrospective study including 22 MEN1 patients with pHPT, ^18^F-FCH PET/CT provided more surgically relevant data regarding the number of pathologic parathyroid glands and their localization than ^99m^Tc-sestamibi scintigraphy in 4/11 patients with initial surgery and in 1/4 patient who underwent second surgery (23). Additional large cohort studies are still necessary to confirm the usefulness of ^18^F-FCH PET/CT in patients with hereditary disorders, such as MEN.

About 2–10% of pHPT patients develop persistent or recurrent disease after first surgery. In those cases, reoperation must be evaluated carefully due to a higher rate of complications than the initial surgery. Typical situations are the presence of unknown ectopic glands, unrecognized MGD, and a negative preoperative imaging workup. Hence, an upfront optimized imaging for the detection and precise localization of hyperfunctioning parathyroid glands remains crucial, as the first intervention is the best time to achieve patient cure. Despite the only limited data available regarding the role of ^18^F-FCH PET/CT in the reoperative setting, ^18^F-FCH PET/CT appears to be a valuable technique to accurately detect hyperfunctioning parathyroid tissue in patients with persistent/recurrent pHPT and is better than 4D-CT and ^99m^Tc-sestamibi scintigraphy (11, 24). In our cohort, ^18^F-FCH PET/CT revealed three hyperfunctioning parathyroid hyperplasia undetected by scintigraphy in two patients with recurrent pHPT. Both cases were cured after surgical excision of the hyperfunctioning parathyroid. Another patient with recurrent pHPT and scinti+/PET- imaging pattern showed persistent disease after the excision of a parathyroid hyperplasia.

A worldwide shortage of Molybdenum-99/Technetium-99 m has been recently experienced with major impact in clinical nuclear medicine practices, reducing the access to broadly performed imaging and diagnostic tests. In this context, ^18^F-FCH PET/CT represents a viable first-line option, ensuring diagnostic and therapeutic continuity in patients with pHPT if ^99m^Tc-sestamibi scintigraphy is not available. On the other hand, the higher costs, the reimbursement and licensing not always possible are potential drawbacks of ^18^F-FCH PET/CT. However, there is currently a lack of data from large patient cohorts with cost-effectiveness evaluation comparing the combination of neck US and parathyroid scintigraphy with ^18^F-FCH PET/CT as a first-line investigation, making it difficult for clinical diagnostic practices to evolve.

The risk of false positive results in patients with inflammatory lymph nodes and/or thyroid nodules is another major potential limitation of ^18^F-FCH PET/CT. Although, its impact on sensitivity is still a matter of debate, the combined use of ^18^F-FCH PET and 4D/CT in one stop-shop examination could potentially help in the differential diagnosis, lowering the incidence of false positive PET/CT results (25–28).

Our study suffers from several limitations that require precisions. First, the limited number of patients due to both the inclusion criteria (pre-PET positive scintigraphy) and the retrospective nature of the investigation. In addition, the long delay between a ^99m^Tc-sestamibi-positive scintigraphy and ^18^F-FCH PET/CT potentially could have affected the results. However, no major changes in biological parameters (PTH, calcemia) were observed during the time from scintigraphy to PET scan, reducing the potential risk of bias. Finally, 6-month follow-up was not achieved for all included patients. However, normal glands (true-negative results) were defined based on a combination of the results of surgical neck exploration, biological follow-up (PTH, serum calcium), and Miami’s “>50% intraoperative PTH decline” criterion, which strength the analysis.

In conclusion, in a preoperative setting, ^18^F-FCH PET/CT appears more accurate and useful than ^99m^Tc-sestamibi scan even in patients with positive scintigraphic results, and may be preferred over ^99m^Tc-sestamibi scan allowing the identification of patients with MGD. A positive parathyroid scintigraphy could not be adequate and sufficient before neck surgery in a not negligible percentage of patients with pHPT and related multiple hyperfunctioning parathyroids, suggesting the need to evolve the practice and define new preoperative imaging algorithms including ^18^F-FCH PET/CT at the fore-front in pHPT patients.

## Data availability statement

The original contributions presented in the study are included in the article/supplementary material, further inquiries can be directed to the corresponding author.

## Ethics statement

The studies involving human participants were reviewed and approved by Ethical Committee of Strasbourg University Hospital and Faculty of Medicine (CE-2022-100). The patients/participants provided their written informed consent to participate in this study.

## Author contributions

AI, JB, GT, and AP: conception or design of the work. AI, JB, GB, AL, CH, UC, MV, GT, and AP: acquisition, analysis, or interpretation of data for the work and drafting the work or revising it critically for important intellectual content. All authors have read and agreed to the submitted version of the manuscript.

## Conflict of interest

The authors declare that the research was conducted in the absence of any commercial or financial relationships that could be construed as a potential conflict of interest.

## Publisher’s note

All claims expressed in this article are solely those of the authors and do not necessarily represent those of their affiliated organizations, or those of the publisher, the editors and the reviewers. Any product that may be evaluated in this article, or claim that may be made by its manufacturer, is not guaranteed or endorsed by the publisher.
